# Cardiopulmonary Response in Post-COVID-19 Individuals: A Cross-Sectional Study Comparing the Londrina Activities of Daily Living Protocol, 6-Minute Walk Test, and Glittre Activities of Daily Living Test

**DOI:** 10.3390/healthcare12070712

**Published:** 2024-03-24

**Authors:** Reem Jasim Al Yammahi, Gopala Krishna Alaparthi, Arthur de Sá Ferreira, Kalyana Chakravarthy Bairapareddy, Fatma A. Hegazy

**Affiliations:** 1 Department of Physiotherapy, College of Health Science, University of Sharjah, Sharjah 27272, United Arab Emirates; u19102377@sharjah.ac.ae (R.J.A.Y.); g.alaparthi@mmu.ac.uk (G.K.A.); kreddy@sharjah.ac.ae (K.C.B.); 2 Department of Health Professions, Faculty Health and Education, Manchester Metropolitan University, Manchester M15 6BX, UK; 3 Postgraduate Program in Rehabilitation Sciences, Augusto Motta University Center (UNISUAM), Rua Dona Isabel 94, Bonsucesso, Rio de Janeiro 21032-060, Brazil; asferreira@unisuam.edu.br; 4 Faculty of Physical Therapy, Cairo University, Cairo 12613, Egypt

**Keywords:** activities of daily living (ADL), cardiopulmonary response, Londrina ADL protocol, Glittre ADL test, 6-minute walk test, post-COVID-19

## Abstract

This study addresses the imperative need for reliable assessment protocols in guiding rehabilitation interventions for individuals post-COVID-19, considering the enduring physiological effects of the virus. A cohort of 40 post-COVID-19 individuals underwent assessments using the Londrina ADL protocol, Glittre ADL test, and the 6-minute walk test (6MWT). Physiological parameters were recorded during and after each test, including heart rate, respiratory rate, and oxygen saturation. The post hoc comparisons between the pre-test and post-test cardiopulmonary response of the three tests showed significant differences, except diastolic blood pressure (6MWT vs. Londrina ADL protocol), heart rate (6MWT vs. Londrina ADL protocol), respiratory rate (6MWT vs. Londrina ADL protocol), blood oxygen level (SpO2) (6MWT vs. Londrina ADL protocol), dyspnea (Londrina ADL protocol vs. Glittre ADL test), and fatigue (Londrina ADL protocol vs. Glittre ADL test). The Londrina ADL protocol demonstrated cardio-pulmonary responses comparable to the Glittre ADL test, as well as the 6MWT, emphasizing its effectiveness in evaluating walking-related outcomes. The study concludes that the Londrina ADL protocol is a robust and practical tool for the routine clinical testing of daily living activities in post-COVID-19 individuals. While the 6MWT remains valuable for assessing walking-related outcomes, a combined approach employing the Londrina ADL protocol and 6MWT offers a comprehensive strategy for evaluating multifaceted functional capacities in this population.

## 1. Introduction

COVID-19 is an extraordinarily contagious respiratory disease. From its initial report in Wuhan, China, it spread worldwide in December 2019 [1]. The primary mode of transmission typically involves the release of airborne droplets when an individual who is infected speaks, coughs, or sneezes. The incubation period of the virus, or how long it takes to go from being infected to showing symptoms, can range from 2 to 14 days [2]. The COVID-19 pandemic has devastated the global population, causing widespread illnesses, death, and economic constraints [3,4], with over 700 million confirmed cases worldwide and over 6 million deaths [5]. COVID-19 has had a significant impact on the United Arab Emirates as well; nearly 1 million verified cases and over 2000 deaths have been documented nationwide [5].

Individuals infected with the SARS-CoV-2 virus exhibit a range of symptoms, such as fever, difficulty breathing, diminished taste, coughing, fatigue, muscle aches, and even a decrease in the sense of smell [6]. COVID-19 is expected to have a significant effect on physical, cognitive, behavioral, and social health status [7], including in patients with mild disease [8]. Furthermore, COVID-19 has been linked to a variety of symptoms, including chronic cardiopulmonary dysfunction, dyspnea, muscle weakness, pain, exhaustion, depression, anxiety, and vocational issues [9]. Thus, it leads to decreased exercise capacity and physical activity, which adversely affects quality of life [10].

Middle-aged and older adults who acquired pulmonary difficulties due to this illness were thought to have a high chance of hospitalization and a severe risk of long-term lung damage [11]. Based on this criterion, healthcare providers and researchers started observing the impact of COVID-19 on individuals infected with mild and severe illnesses. According to insights provided by Pizarro-Pennarolli et al. [12], the effects of COVID-19 on the ability to perform activities of daily living (ADL)—e.g., getting dressed, using the restroom, and moving from one location to another—may diminish a person’s functional capability. The fatigue, shortness of breath, and body soreness caused by COVID-19 might make it difficult for individuals to carry out their everyday activities properly [12]. Fatigue, for instance, is the most described post-COVID-19 symptom and is linked to a notable decline in physical, cognitive, and emotional functioning that may deteriorate ADLs [13,14]. Therefore, the evaluation of ADL and the analysis of cardiopulmonary responses during the assessment can help in the development of an effective recovery program for patients with COVID-19.

The 6-minute walk test (6MWT) is an objective, acceptable, and standardized test that assesses functional exercise capacity in patients with chronic lung illness in outpatient primary and pulmonary practices [15,16,17]. In 2002, the American Thoracic Society released a recommendation statement on the application of the 6MWT in clinical practice [18]. The psychometric assessment of the 6MWT demonstrated good validity and reliability among individuals diagnosed with chronic obstructive pulmonary disease (COPD) [19,20]. Furthermore, Ferioli et al. [16] and Modi et al. [21] applied the 6MWT on patients post-COVID-19 and concluded that the 6MWT is a useful test for measuring functional capacity in this population [11].

The Glittre ADL test, on the other hand, involves activities of both upper and lower limbs and other tasks such as ascending and descending steps, sitting and rising from a chair, and arm motions when holding weights in addition to walking [22]. The Glittre ADL test was one of the measures suggested by Deshpande et al. [22] and Mendes et al. [23] for evaluating ADL. They tested patients with COPD with the Glittre ADL test and found that it is a useful tool for evaluating functional performance and ADL in patients with COPD. The Glittre ADL test tends to be a reliable and more appropriate choice for evaluating an individual’s functional ability than the 6MWT [23]. The 6MWT and Glittre ADL test, as mentioned above, focus to some extent on the upper limbs, but mainly on the lower limbs’ activities, but these tests do not involve psychological or cognitive state of mind, which has an important role in the performance of ADL. The Londrina ADL protocol involves the assessment of psychological or cognitive factors in achieving the goals of ADL [24]. Studies performed on COPD subjects concluded that the Londrina ADL protocol is a valid and reliable protocol for evaluating ADL in patients with COPD [24].

There is a lack of evidence on investigating the cardiopulmonary response induced by the Londrina ADL protocol in people with post-COVID-19. This study seeks to fill the existing gap in the literature by thoroughly examining the cardiopulmonary reaction triggered by the Londrina ADL protocol in individuals recovering from COVID-19 and comparing it to the responses elicited by the 6MWT and the Glittre ADL test. Given the persisting cardiovascular and respiratory symptoms observed in post-COVID-19 patients, understanding the physiological demands of daily activities becomes crucial for effective rehabilitation. The choice of the Londrina ADL protocol is strategic, as it offers a detailed and context-specific assessment of functional capacity [24]. By comparing the cardiopulmonary response induced by this protocol with established measures such as the 6MWT and the Glittre ADL test, we seek to portray the unique insights provided by the Londrina ADL protocol. This comparative analysis helps to identify the most sensitive and specific tool for evaluating cardiopulmonary function in post-COVID-19 individuals, informing tailored rehabilitation strategies and contributing to the optimization of patient care in the aftermath of the pandemic. Ultimately, this research is poised to enhance our understanding of the cardiopulmonary implications of post-COVID-19 syndrome, offering valuable implications for clinical practice and public health interventions.

## 2. Materials and Methods

### 2.1. Study Design and Setting

This cross-sectional study employed a comparative design to investigate the cardiopulmonary response in individuals with post-COVID-19 using three different functional assessment protocols: the Londrina ADL protocol, the 6-minute walk test (6MWT), and the Glittre ADL test, all of which are described in detail in the upcoming sections.

### 2.2. Participants

This study included a sample of adult individuals with confirmed post-COVID-19 status, determined through medical records and diagnostic criteria established by health authorities. Participants were recruited from rehabilitation centers, outpatient clinics, and community health settings in the Sharjah region, UAE.

#### 2.2.1. Inclusion Criteria

To be included in this study, individuals had to meet several criteria. First, individuals aged between 18 and 65 years and who had a confirmed diagnosis of COVID-19 through laboratory testing were included. Furthermore, participants who had post-COVID-19 mild to moderate disease severity, with mild conditions defined by a COVID-19 severity index score of 0–2 and moderate conditions indicated by a score of 3–5, as outlined by Huespe et al. [25]. Additionally, prospective participants had to demonstrate the ability to comprehend and follow provided instructions, reflecting a basic level of cognitive understanding.

#### 2.2.2. Exclusion Criteria

Several exclusion criteria were established for this study. Firstly, individuals with unstable cardiovascular or respiratory conditions requiring long-term oxygen therapy were not considered for participation. Those diagnosed with COVID-19 and presenting severe symptoms, as defined by the COVID-19 severity index [25], were also excluded. Severe symptoms encompass signs of pneumonia and specific criteria such as a respiratory rate exceeding 30 breaths per minute, severe respiratory distress, or oxygen saturation levels falling below 90% in ambient air. Additionally, individuals with known cardiovascular conditions significantly impacting functional capacity, for example, recent myocardial infarction, were not eligible for inclusion. Participants with confirmed severe cognitive or mental impairment, as diagnosed by a medical professional, were excluded from the study. Prospective participants unable to perform functional assessments due to musculoskeletal limitations, such as fractures or severe osteoarthritis, were also excluded. Finally, individuals with prosthetic devices or orthoses were excluded from this study.

### 2.3. Sample Size

The sample size was determined a priori using G*Power 3.1.9.6 [26]. We used Pearson’s correlation coefficient for estimating the correlation between the tests’ outcomes as an estimate of minimal construct validity between tests. Given a 5% type-I error (two-tailed), 20% type-II error, and a correlation between outcomes equal to or higher than 0.4, similar to that reported by Deshpande et al. [22], a minimum of 46 participants is required.

### 2.4. Ethical Considerations

The study protocol has been approved by the Institutional Review Board of the researchers’ institution, ensuring compliance with ethical standards for human research. Informed consents were obtained from each participant before their involvement in the study. After receiving approval from the Research Ethics Committee of the University of Sharjah [REC-21-06-23-01-S], data collection was initiated.

### 2.5. Outcome Measures

This research utilized a comprehensive set of outcome measures to assess various dimensions of health and functional ability of individuals recovering from COVID-19. These three outcome measures are described in the following section.

#### 2.5.1. The 6-Minute Walk Test (6MWT)

The 6MWT serves as a pivotal metric, evaluating participants’ exercise capacity and endurance by measuring the total distance covered walking over a six-minute period. This objective measure provides valuable insights into the overall physical fitness and mobility of the study cohort. Participants were advised to walk ‘as far as’ possible for 6 min along a 30 m corridor to cover their entire walking distance (measured in meters) in that time. Every minute, standardized instructions were given. Participants were allowed to pause and rest, if necessary, using the phrase: “You can lean against the wall if you would like; then continue walking whenever you feel able.” At the start and immediately after the procedure, blood pressure (BP), heart rate (HR), respiratory rate (RR), oxygen saturation (SpO2), and dyspnea (the modified Borg scale) were calculated. Fatigue was measured using the modified Borg Rating of Perceived Exertion scale. The modified Borg scale is a widely used tool for assessing perceived exertion or fatigue during physical activity [27]. This scale offers a simple yet effective method for quantifying fatigue levels, making it valuable in various fields such as sports medicine, rehabilitation, and clinical research [28]. The 6MWT was performed twice on subsequent days, and the best value was recorded [15,16]. 

#### 2.5.2. The Glitter Activities of Daily Living (ADL) Test

The Glittre ADL test involves the physical performance of nine daily activities [23]. It requires walking a 10 m circuit carrying backpacks weighing 2.5 kg and 5 kg for female and male participants, respectively. The 10 m circuit requires the patient to change from sitting to standing position before walking a flat course. The circuit includes a two-step staircase measuring 17 cm by 27 cm in the middle of the course. The second station involves moving 1 kg of objects from a shoulder-high shelf to a waist-high shelf. The 1 kg objects are moved from the shelves, beginning with the lower shelf and then the top shelf [22]. The participants return to the 10 m circuit again by descending the two-step staircase in the middle of the course. The 10 m circuit ends with a chair where the participant sits before the next lap [29]. Subjects were instructed to complete 5 laps ‘as fast as possible’. A stopwatch was used to measure the time it took to complete the test at the end. Patients were advised to perform the tasks at their own speed. Before beginning the protocol, the evaluator demonstrated the activities one by one. BP, RR, HR, sensation of dyspnea (the modified Borg scale), and SpO2 were measured at the beginning and immediately after the test. The Glittre ADL test was performed twice on subsequent days, and the best value was taken [29]. The Glittre ADL test serves as a dynamic evaluation tool, focusing on activities that simulate real-life scenarios. This test is particularly valuable in assessing functional capacity by replicating daily tasks in a controlled environment. By incorporating dynamic movements, the Glittre test provides a nuanced perspective on participants’ ability to perform activities essential to daily living, offering insights beyond traditional static assessments.

#### 2.5.3. The Londrina Activities of Daily Living Protocol

The Londrina ADL protocol is employed to assess participants’ functional independence in their daily lives. This protocol examines daily activities, offering a nuanced understanding of how individuals navigate and accomplish routine tasks [24]. By capturing the activities of daily living, this measure contributes to a holistic assessment of participants’ functional abilities beyond simple endurance. The protocol was performed as follows:

Objects on the table: The participant sits in a chair in front of a table with a line dividing it into two halves (dimensions: 120 cm [length] 60 cm [width]) (left and right). The table has ten objects on it, all of which are on the left half of the table (4 objects weighing 250 g, 4 objects weighing 500 g, and 2 objects weighing 1 kg each). In both hands, the participant picks up the items one by one and positions them all on the table. The participant then returns all of the objects to the right side of the table in the same manner.

Walking with bags: The participant walks across a 6 m line three times in a row (back and forth for a total of 18 m) while holding two bags, one in each hand. Loads comprising 10% of the subject’s body weight are found inside the bags (5 percent in each bag).

Shelves: The subject stands in front of four shelves, one above the other (42 cm between the floor and the first shelf; 45 cm between one shelf and the next), with a table beside them. There are 12 items on the table (4 objects of 250 g, 4 objects of 500 g, 2 objects of 1 kg, and 2 objects of 2 kg). Using both hands, the individual picks up the items one by one and puts them on the shelves (without a predetermined order). The subject arranges the items on the shelves such that three of them are on each shelf. After putting all of the items on the shelves, the subject returns them to the table in the same manner (without a predetermined order).

Clothesline: The subject stands in front of an eye-level clothesline. A basket containing ten pieces of clothing (median weight of items = 122 g [range 80–442 g]) is next to the subject on the ground. With both hands, the subject takes all of the things and hangs them on the clothesline one by one. After hanging all of the clothes, the subject puts them back in the basket one at a time, with both hands.

Walking: The subject walks back and forth three times on the same 6 m line as in activity 2, but without holding any bags.

Participants were instructed to perform these tasks as if they were doing them at home, at their regular day-to-day pace. If they needed rest, they were free to rest, and no motivation was provided while applying the test. Before beginning the protocol, the evaluator demonstrated the activities one by one. BP, HR, RR, SpO2, and sensation of dyspnea (mod Borg scale) were measured at the beginning and immediately after the test. The Londrina protocol was performed twice on subsequent days, and the best value was taken. The validity and reliability of the Londrina ADL protocol have been established to evaluate ADL performance in individuals with COPD [22,24].

Before the initiation of the program, all participants underwent an assessment of their demographics (e.g., age, gender), pulmonary function, and anthropometric measurements. Lung function was evaluated using an EasyOne spirometer from NDD Medical Technologies in Zurich, Switzerland, and its calibration was verified before each assessment. Spirometry procedures adhered to the standards set by the American Thoracic Society/European Respiratory Society [30]. Forced vital capacity (FVC) and forced expiratory volume in one second (FEV1) were measured in liters and as a percentage of the predicted value (%pred). Absolute and percent reference values for FEV1, FVC, FEV1/FVC, and PEFR were calculated based on the Global Lung Function Initiative (GLI) [31]. The vital signs were measured using a pulse oximeter (Oxiline Pulse XS Pro, Miami, FL, USA) and sphygmomanometer (Omron Hem 7120, Omron Manufacturer, Vietnam), of which the validity and reliability have been well established [32,33].

### 2.6. Study Procedure

#### 2.6.1. Sampling Method

The study employed a convenience sampling method, where participants meeting the inclusion criteria were recruited from a pool of eligible individuals. Once the study protocol was approved by the scientific committee and research ethics committee in the University of Sharjah, the participants were invited to participate in the study according to the inclusion/exclusion criteria outlined above. The study was explained to the participants with mild and moderate post-COVID-19, who were referred to by a doctor or pulmonologist. After signing a written consent form, willing participants were screened for the inclusion and exclusion criteria.

#### 2.6.2. Sequence of Tests

Participants underwent a structured sequence of assessments. Firstly, all subjects had their pulmonary function and anthropometric measurements evaluated. Then, they completed the 6MWT to evaluate functional capacity. Subsequently, the Londrina ADL protocol was administered to assess functional independence in routine activities. Finally, the Glittre ADL test was conducted to evaluate dynamic functional capacity. All the eligible participants completed the three tests, twice each on separate days. No randomization was performed as carry-over or learning effects are unlikely to happen due to the washout period and characteristics of the tests.

Washout Period: To minimize potential carryover effects between tests, a standardized washout period of one hour was implemented. This interval allowed participants to return to baseline conditions before undertaking subsequent assessments, ensuring the independence of the test results.

Study Setting: All assessments were conducted in a controlled and standardized environment to ensure consistency. The study setting was a dedicated research facility equipped with the necessary infrastructure for administering the tests.

Investigator and Outcome Recording: A trained and qualified investigator conducted the tests. The investigator was responsible for guiding participants through the assessments, providing instructions, and ensuring adherence to the standardized procedures. Additionally, outcomes were systematically reviewed by a designated research supervisor who was not involved in the direct administration of the tests. This separation of roles helped maintain objectivity and reduce potential bias in outcome recording.

By employing this structured approach, including a convenient sampling method, a well-defined sequence of tests, a washout period, and a controlled study setting with designated roles for investigators and outcome recorders, the study aimed to ensure the reliability and validity of the collected data.

### 2.7. Statistical Analysis

The data analysis was conducted using IBM SPSS Statistics for Windows, ver. 25.0. Armonk, NY, USA, IBM Corp. Initially, descriptive statistics were employed to characterize the study sample based on their demographic features. Continuous variables were presented as mean ± standard deviation, while categorical variables were expressed as frequencies and percentages. Subsequently, the normality of the data was assessed using the Shapiro–Wilk test. Paired sample *t*-tests were utilized to compare differences in the cardio-pulmonary response before and after each test. An independent repeated-measures analysis of variance (ANOVA) was applied to compare each cardio-pulmonary response outcome among tests with a post hoc analysis for a paired comparison. The significant *p*-value cutoff score was set at 0.05.

## 3. Results

A total of 46 post-COVID-19 individuals were initially recruited for the study. However, six participants were excluded based on predetermined eligibility criteria. Exclusion factors included two participants experiencing breathlessness during testing, three reporting dizziness, and one declining to participate due to fatigue. Following exclusions, the final study cohort consisted of 40 participants. As shown in Table 1, the gender distribution within the retained sample was balanced, with females comprising 52.5% and males 47.5% of the cohort. The mean age of the participants was 37.00 years, with a standard deviation of 8.70. Anthropometric measurements revealed a mean height of 167.05 cm (standard deviation = 8.70), mean weight of 74.23 kg (standard deviation = 12.64), and a corresponding mean body mass index (BMI) of 26.45 (standard deviation = 2.69). Furthermore, the distribution of COVID-19 severity within the retained cohort was characterized by 80% of individuals having experienced mild disease and 20% with a history of moderate disease. These demographic and clinical characteristics provide a comprehensive overview of the study population, setting the stage for the subsequent analyses of post-COVID-19 cardiopulmonary responses.

The pulmonary function of the study participants was assessed through a series of measurements, yielding the following results. The mean forced vital capacity (FVC) was found to be 3.60 L, with a standard deviation of 0.88, indicating the average maximum volume of air forcefully exhaled after a maximal inhalation. The forced expiratory volume (FEV1), representing the volume of air expelled during the first second of the FVC maneuver, exhibited a mean of 2.95 L, with a standard deviation of 0.71. Peak expiratory flow, a measure of the maximum flow rate during forced expiration, demonstrated a mean of 6.17 L per second, with a standard deviation of 1.90. Additionally, the forced expiratory volume to forced vital capacity ratio (FEV1/FVC) exhibited a mean of 0.82, with a standard deviation of 0.08, providing insights into the proportion of vital capacity expelled in the first second. These pulmonary function test results collectively contribute to the comprehensive assessment of respiratory health in the post-COVID-19 individuals under study (see Table 1).

Table 2 presents a comparison of pre- and post-cardiopulmonary responses in the 6-minute walk test (6MWT) among post-COVID-19 participants. The analysis revealed statistically significant differences in several key parameters. Notably, systolic blood pressure, diastolic blood pressure, heart rate, respiratory rate, and fatigue (measured on the modified Borg scale) exhibited significant changes between pre- and post-6MWT assessments, with *p*-values less than 0.001.

In contrast, no significant difference was observed in blood oxygen saturation (SpO2) levels (*p* = 0.70) post-6MWT, indicating that the participants maintained consistent oxygen saturation levels during the test. However, it is noteworthy that dyspnea showed a statistically significant difference (*p* = 0.04), suggesting alterations in perceived breathlessness following the 6MWT.

Table 3 presents a detailed comparison of pre- and post-cardiopulmonary responses in the Londrina ADL protocol test among post-COVID-19 participants. The analysis revealed statistically significant differences in several key parameters.

Significant alterations were observed in systolic and diastolic blood pressure, heart rate, respiratory rate, dyspnea, and fatigue (*p* < 0.001) post-Londrina ADL protocol test. However, no significant difference was found in blood oxygen saturation (SpO2) levels (*p* = 0.61) post-Londrina test, indicating a maintained oxygen saturation during the simulated daily living activities.

Table 4 provides a comparison of pre- and post-cardiopulmonary responses in the Glittre ADL test among post-COVID-19 participants. The analysis reveals statistically significant differences in all the variables examined. Significant changes were observed in systolic and diastolic blood pressure, heart rate, respiratory rate, dyspnea, fatigue, and blood oxygen saturation (SpO2) (*p* < 0.001) post-Glittre ADL test.

Table 5 outlines the post hoc comparisons between the pre-test and post-test cardiopulmonary response of the three tests (6-minute walk test, Londrina ADL protocol, and Glittre ADL test) in post-COVID-19 subjects, showing varied results. All variables show significant differences between the pre- and post-test results, except diastolic blood pressure (6MWT vs. Londrina ADL protocol), heart rate (6MWT vs. Londrina ADL protocol), respiratory rate (6MWT vs. Londrina ADL protocol), blood oxygen level (SpO2) (6MWT vs. Londrina ADL protocol), dyspnea (Londrina ADL protocol vs. Glittre ADL test), and fatigue (Londrina ADL protocol vs. Glittre ADL test).

## 4. Discussion

### 4.1. The Main Findings

This study explored the cardiopulmonary response triggered by the Londrina activities of daily living protocol in 40 individuals who had recovered from COVID-19 in Sharjah, UAE. The study compared this response with the effects observed during the 6MWT and Glittre ADL test. The findings indicated significant alterations in the cardiopulmonary response, including physiological changes in heart rate, respiratory rate, and systolic and diastolic blood pressure, following the performance of both the Glittre ADL test and Londrina ADL protocol. In contrast, the 6MWT yielded a milder physiological response compared to the other two tests. Additionally, participants reported experiencing a lower level of dyspnea (*p* = 0.04) during the 6MWT in comparison to the Londrina ADL protocol and Glittre ADL test.

These findings can be explained in part by the fact that both the Londrina ADL protocol and Glittre ADL test entail upper extremity tasks, whereas the 6MWT only requires lower limb performance. During the upper extremity tasks, extra demands are placed on the accessory respiratory muscles, which affect ventilation and cause extra burden to the diaphragm, leading to dyssynchronous thoracoabdominal breathing movements that are associated with the onset of severe dyspnea [34].

In a comparable investigation conducted by Deshpande et al. [22] on individuals with COPD, the study compared the cardiopulmonary responses of the 6MWT to the Glittre ADL test and Londrina ADL protocol. The findings indicated that subjects exhibited a higher cardiopulmonary response to the Glittre ADL test and Londrina ADL protocol compared to the 6MWT [22]. The results of our current study align with the research conducted by Cavalheri et al. [35], who focused on COPD individuals undergoing the Glittre ADL test. Their findings suggested that activities such as climbing stairs resulted in a higher energy expenditure compared to walking, leading to increased dyspnea and fatigue [35]. Conversely, tasks involving moving objects on and off a shelf without arm support required the least energy [22]. However, a study by Karloh et al. [29] revealed that a significant portion (50–65%) of one lap in the Glittre ADL test was spent on moving objects without hand support, resulting in an elevated metabolic demand. Similarly, Velloso et al. [36] and Gulrat et al. [37] demonstrated that individuals with COPD utilized a substantial portion of their ventilatory reserve during ADLs, contributing to ventilatory limitations in their functional capacity.

Furthermore, there is documented evidence indicating that the Londrina ADL protocol can induce changes in cardiopulmonary responses, even in healthy individuals. This claim was confirmed by a study conducted in India by Sreedevi et al. [38] on data collected using the Londrina ADL protocol from healthy individuals aged 40–60 years. The study affirms that the Londrina ADL protocol is a reliable method for assessing ADLs, revealing a slight increase in heart rate, respiratory rate, dyspnea, and fatigue in healthy subjects both before and after the test. This phenomenon may be attributed to the diverse range of activities included in the Londrina ADL protocol, each demanding a different level of energy expenditure [24]. Sreedevi et al.’s study propose that the observed increase in physiological parameters could be a result of heightened metabolic demand during exercise, leading to an elevated cardiac output to meet peripheral oxygen requirements [38].

Another potential reason for the enduring fatigue, dyspnea, and ventilatory limitations following COVID-19 could be linked to symptoms like myalgia, which has been observed in approximately 15% of patients even four months after recovering from the infection [39]. While ongoing research is still investigating the extent of the sequelae resulting from the infection at various functional levels, persisting effects such as fatigue and dyspnea have been documented beyond hospital discharge, with the long-term impact on different functional levels remaining unclear [40,41]. Consequently, it appears essential and logical to enhance the clinical evaluation of physical capacity by including a comprehensive analysis of functional abilities related to the performance of daily activities in this population.

The comparison of cardiopulmonary responses before and after testing across the three distinct assessments—the 6MWT, Londrina ADL protocol, and Glittre ADL test—in post-COVID-19 participants revealed noteworthy findings. The observed significant differences in several variables underscore the impact of post-COVID-19 sequelae on cardiopulmonary functions. For example, the 6MWT, a widely used measure of exercise capacity, demonstrated significant changes in various parameters. However, when compared to the Londrina ADL protocol, no significant differences were observed in diastolic blood pressure, heart rate, respiratory rate, and blood oxygen level (SpO2). This suggests that the two tests may tap into different aspects of cardiopulmonary response, highlighting the importance of employing multiple assessments to comprehensively evaluate post-COVID-19 individuals.

Interestingly, the comparison between the Londrina ADL protocol and the Glittre ADL test revealed no significant differences in dyspnea and fatigue. Deshpande et al. [22] conducted a comparative study between the Londrina ADL protocol and Glittre ADL test on a sample of individuals with COPD and reported similar results. These findings suggest that these two measures, focusing on daily living activities, may capture similar aspects of functional impairment in post-COVID-19 individuals. The lack of significant differences in these specific variables indicates potential overlap in the constructs measured by these assessments.

### 4.2. Clinical Implications of the Findings

The study of cardiopulmonary responses in post-COVID-19 individuals holds significant implications for rehabilitation professionals in the assessment and planning of treatment strategies. The nature of the assessments utilized in this study offers a multifaceted understanding of post-COVID-19 functional limitations. Clinicians can leverage the insights gained from these cardiopulmonary assessments to tailor rehabilitation interventions based on individualized needs. The identification of specific parameters that exhibit significant changes provides a targeted approach for addressing post-COVID-19 sequelae. For example, understanding the unique challenges presented by dyspnea and fatigue in daily living activities, as revealed by the Londrina ADL protocol and Glittre ADL test, allows for the development of focused interventions to improve functional capacity and quality of life. Furthermore, the comparison with previous studies enriches the evidence base for post-COVID-19 rehabilitation strategies. Consistent findings across studies may corroborate the reliability of certain assessments, while divergent results can stimulate further inquiry into the underlying mechanisms of cardiopulmonary responses in the post-COVID-19 population.

Overall, this research contributes to the evolving landscape of post-COVID-19 rehabilitation by elucidating the unique aspects of cardiopulmonary responses. Physiotherapists can utilize these findings to enhance the precision of assessments and tailor interventions that address the specific needs of individuals recovering from COVID-19, ultimately facilitating a more effective and personalized approach to rehabilitation.

### 4.3. Limitation of the Study

Despite providing valuable insights into post-COVID-19 cardiopulmonary responses, this study is subject to some limitations. The sample size may limit generalizability, and the population’s lack of diversity in demographics, disease severity, and comorbidities may impact broader applicability. The cross-sectional design hinders the establishment of causal relationships, and the chosen assessments may not encompass the entirety of post-COVID-19 sequelae, including unaccounted confounders, such as medication use and lifestyle factors, further challenging the interpretation of the results. Despite the washout period, we cannot rule out the order and fatigue effects of the applied assessments. We did not include a control group primarily due to ethical considerations, as healthy individuals would be unnecessarily exposed to tests for the sole purpose of comparison. However, to enhance the impact and informativeness of the study, prospective or historical control group comparisons could be incorporated in future studies to provide insights into the relative effect of the assessments compared to patients without COVID-19. Recognizing these limitations is crucial for a nuanced understanding of the study’s findings, emphasizing the need for future research to address these constraints and enhance the robustness of insights into post-COVID-19 cardiopulmonary responses.

## 5. Conclusions

Our findings underscore the crucial role of tailored assessment protocols in evaluating individuals recovering from post-COVID-19. The Londrina ADL protocol proves invaluable, exhibiting a cardio-pulmonary response comparable to the established Glittre ADL test. Despite slightly weaker responses from the 6MWT, its significance in assessing walking-related outcomes persists. These findings emphasize the necessity of employing comprehensive evaluation methods to gain a nuanced understanding of post-COVID-19 functional capacities. The Londrina ADL protocol, suitable for the routine clinical testing of daily living activities, offers a promising avenue for healthcare practitioners to assess recovery and customize rehabilitation strategies effectively. This research contributes essential insights into the evolving post-COVID-19 care landscape, stressing the importance of diverse assessment tools to capture multifaceted physiological responses in this population.

## Figures and Tables

**Table 1:** Demographics and anthropometric characteristics of the overall sample (*n* = 40).

Characteristic	Mean ± SD
Gender (N%)	
Female	21 (52.5%)
Male	19 (47.5%)
Age, years	37.00 ± 8.70
Height, cm	167.05 ± 8.70
Weight, kg	74.23 ± 12.64
Body mass index, kg/m^2^	26.45 ± 2.69
COVID severity (N%)	
Mild	32 (80%)
Moderate	8 (20%)
**Pulmonary function test**	
Forced vital capacity	
L	3.61 ± 0.88
% predict	93 ± 16
Forced expiratory volume	
L	2.95 ± 0.72
% predict	90 ± 12
Peak expiratory flow	
L/s	6.17 ± 1.90
% predict	79 ± 21
Forced expiratory volume/forced vital capacity	
Ratio	0.83 ± 0.09
% predict	101 ± 11

**Table 2:** Comparing pre- and post-cardiopulmonary response in 6-minute walk test in post-COVID-19 subjects.

Variables	Pre (Mean ± SD)	Post (Mean ± SD)	Mean Difference	*p* Value
SBP	117.27 ± 10.47	121.37 ± 10.84	−4.10	<0.001
DBP	79.10 ± 8.397	84.96 ± 8.593	−5.86	<0.001
HR	80.05 ± 7.21	89.58 ± 7.36	−9.53	<0.001
RR	20.07 ± 1.42	22.37 ± 2.20	−2.30	<0.001
SpO2	98.55 ± 1.176	98.62 ± 0.86	−0.07	0.70
Dyspnea (mod Borg)	0.05 ± 0.22	0.15 ± 0.48	−0.10	0.04
Fatigue (mod Borg)	0.05 ± 0.22	1.25 ± 1.17	−1.20	<0.001
Distance	-	459.10 ± 100.72		

*p* < 0.05 is considered statistically significant, SBP: systolic blood pressure, DBP: diastolic blood pressure, HR: heart rate, RR: respiratory rate, SpO2: blood oxygen level.

**Table 3:** Comparing pre- and post-cardiopulmonary response in Londrina ADL protocol in post-COVID-19 participants.

Variables	Pre (Mean ± SD)	Post (Mean ± SD)	Mean Difference	*p* Value
SBP	119.12 ± 12.87	125.08 ± 13.52	−5.95	<0.001
DBP	79.10 ± 8.39	85.03 ± 9.02	−5.93	<0.001
HR	77.45 ± 8.37	98.27 ± 10.62	−20.82	<0.001
RR	16.68 ± 1.80	23.82 ± 2.57	−7.14	<0.001
SpO2	98.42 ± 0.98	98.35 ± 0.86	0.07	0.61
Dyspnea (mod Borg)	0.05 ± 0.22	0.50 ± 0.64	−0.45	<0.001
Fatigue (mod Borg)	0.07 ± 0.26	3.50 ± 1.82	−3.42	<0.001
Time (s)	-	347.20 ± 30.58		

*p* < 0.05 is considered statistically significant, SBP: systolic blood pressure, DBP: diastolic blood pressure, HR: heart rate, RR: respiratory rate, SpO2: blood oxygen level.

**Table 4:** Comparing pre- and post-cardiopulmonary response in Glittre ADL test in post-COVID-19 subjects.

Variables	Pre (Mean ± SD)	Post (Mean ± SD)	Mean Difference	*p* Value
SBP	119.12 ± 12.87	126.03 ± 13.62	−6.90	<0.001
DBP	79.10 ± 8.39	86.24 ± 8.70	−7.14	<0.001
HR	80.05 ± 7.21	102.10 ± 14.76	−22.05	<0.001
RR	16.68 ± 1.80	27.40 ± 2.96	−10.72	<0.001
SpO2	98.42 ± 0.98	93.66 ± 1.15	4.76	<0.001
Dyspnea (mod Borg)	0.05 ± 0.22	0.47 ± 0.64	−0.42	<0.001
Fatigue (mod Borg)	0.07 ± 0.267	3.35 ± 1.88	−3.27	<0.001
Time (s)		347.20 ± 30.58		

*p* < 0.05 is considered statistically significant, SBP: systolic blood pressure, DBP: diastolic blood pressure, HR: heart rate, RR: respiratory rate, SpO2: blood oxygen level.

**Table 5:** Cardiopulmonary response between 6-minute walk test, Londrina ADL protocol, and Glittre ADL test in post-COVID-19 participants.

Variables	Between the Groups (Post Only)	Mean Difference	*p* Value
SBP	6MWT vs. Londrina ADL protocol	−3.70	<0.001
	6MWT vs. Glittre ADL test	−4.65	<0.001
	Londrina ADL protocol vs. Glittre ADL test	−0.95	<0.001
DBP	6MWT vs. Londrina ADL protocol	−0.06	0.51
	6MWT vs. Glittre ADL test	−1.28	<0.001
	Londrina ADL protocol vs. Glittre ADL test	−1.21	<0.001
HR	6MWT vs. Londrina ADL protocol	3.82	0.20
	6MWT vs. Glittre ADL test	12.52	<0.001
	Londrina ADL protocol vs. Glittre ADL test	8.70	<0.001
RR	6MWT vs. Londrina ADL protocol	−1.45	0.007
	6MWT vs. Glittre ADL test	−5.03	<0.001
	Londrina ADL protocol vs. Glittre ADL test	−3.58	<0.001
SpO2	6MWT vs. Londrina ADL protocol	0.27	0.06
	6MWT vs. Glittre ADL test	4.96	<0.001
	Londrina ADL protocol vs. Glittre ADL test	4.69	<0.001
Dyspnea	6MWT vs. Londrina ADL protocol	−0.35	<0.001
	6MWT vs. Glittre ADL test	−0.32	<0.001
	Londrina ADL protocol vs. Glittre	0.02	0.32
Fatigue	6MWT vs. Londrina ADL protocol	−2.25	<0.001
	6MWT vs. Glittre ADL test	−2.10	<0.001
	Londrina ADL protocol vs. Glittre ADL test	0.15	0.27

*p* < 0.05 is considered statistically significant, SBP: systolic blood pressure, DBP: diastolic blood pressure, HR: heart rate, RR: respiratory rate, SpO2: blood oxygen level.

## Data Availability

The research data will be available on responsible requests.
